# Innovative continuous-irrigation approach for wound care after deep neck infection surgery: A case report

**DOI:** 10.1016/j.ijscr.2021.02.006

**Published:** 2021-02-05

**Authors:** Meng-Chang Ding, Chih-Yuan Lee, Yun-Ting Wang, Cheng-Ming Hsu, Yao-Te Tsai, Ming-Shao Tsai

**Affiliations:** a Department of Otolaryngology – Head and Neck Surgery, Chiayi Chang Gung Memorial Hospital, Chiayi, Taiwan; b Nursing Department, Chiayi Chang Gung Memorial Hospital, Chiayi, Taiwan; c Graduate Institute of Clinical Medical Sciences, Chang Gung University, Taoyuan, Taiwan; d College of Medicine, Chang Gung University, Taoyuan, Taiwan

**Keywords:** Irrigation, Neck, Cervical, Cellulitis, Abscess

## Abstract

**Introduction:**

Deep neck infection is a life-threatening disease that invades deep neck space and potentially causes airway obstruction. Treatment for deep neck infection consists of antibiotic administration and surgical drainage with manually postoperative wound irrigation. We herein present a case in which an innovative continuous-irrigation approach was applied for wound care following surgical drainage.

**Presentation of case:**

A 65-year-old woman presented with neck swelling and fever for 5 days. Computed tomography of the head and neck revealed a deep neck infection with abscess formation. The patient underwent surgical incision and drainage of the deep neck abscess. We employed an innovative continuous-irrigation approach for wound care after surgery using a double-lumen tube consisting of an inlet tube and an outlet tube. Saline water was continuously injected through the irrigation tube and suctioned from the draining tube. After 5 days of intensive irrigation, wound swelling and discharge was considerably reduced, and the wound had been closed.

**Discussion:**

This patient with deep neck infection was successfully treated using an innovative continuous-irrigation approach for wound care after surgery. This approach exhibited several advantages. First, compared with intermittently manual irrigation, a continuous-irrigation device can more effectively keep a wound clean. Second, the automated design of this device can reduce the workload for clinical staff. Third, our device does not require expensive materials or complex technology.

**Conclusion:**

This innovative continuous-irrigation approach is an alternative approach for wound care in patients with deep neck infection.

## Highlights

• Deep neck infection is a life-threatening disease that invades deep neck space.
• Treatment for deep neck infection consists of antibiotics and surgical drainage with manually postoperative wound irrigation.
• The authors present a case in which an innovative continuous-irrigation approach was applied for wound care.
• This approach is an alternative approach for wound care in patients with deep neck infection.

## 1 Introduction

Deep neck infection is a dangerous and life-threatening disease that invades deep neck space and potentially causes airway obstruction [1,2]. Treatment for deep neck infection mainly consists of antibiotic administration and surgical drainage with postoperative wound irrigation [3]. Postoperative irrigation should be performed every 4–8 hours by doctors according to disease severity, which imposes additional physical and time-related burdens on medical staff [[4], [5], [6]]. Herein, we present a case in which an innovative continuous-irrigation approach was applied for wound care following surgery for deep neck infection. This case is reported in accordance with the SCARE 2020 guideline [7].

## 2 Presentation of case

**Fig. 1:**
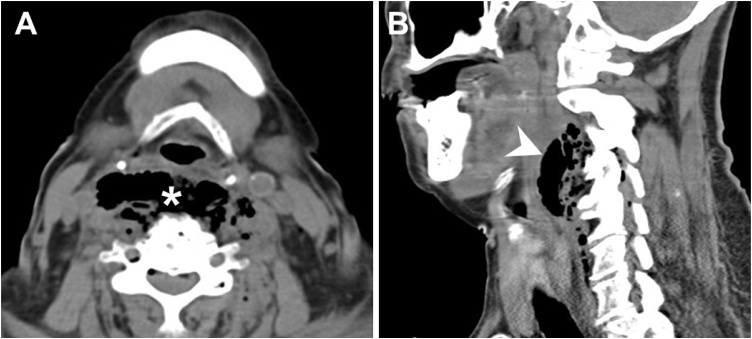
A 65-year-old female patient with deep neck infection. (A) Axial view of the computed tomography image revealing gas collection in the bilateral retropharyngeal, parapharyngeal, and carotid spaces (asterisk). (B) Sagittal view of the computed tomography image presenting diffuse soft-tissue swelling with fluid and air collection in the prevertebral region (arrowhead).

**Fig. 2:**
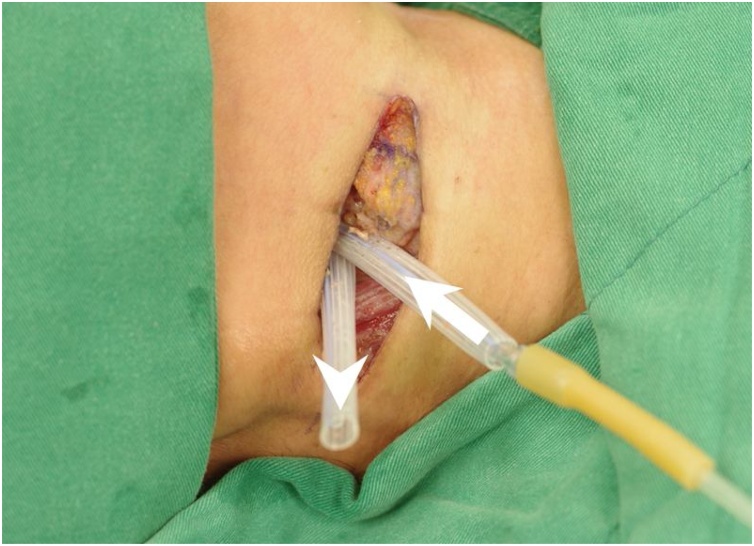
Innovative continuous-irrigation approach for wound care. The device consists of an input tube (arrow) and a discharge tube (arrowhead). The input tube is connected to a continuous normal saline drip, and the discharge tube is connected to a low-pressure suction device for drainage.

The Institutional Review Board of Chang Gung Memorial Hospital approved this study (CGMH-IRB No. 202002100B0). Patient has provided informed consent for publication of the case. A 65-year-old woman presented with neck swelling and reported having a fever for 5 days and progression to dysphagia and dyspnea in the past 1 day. She came to the emergency room, where computed tomography of the head and neck revealed an abscess and gas accumulation in the bilateral retropharyngeal, parapharyngeal, and carotid spaces (Fig. 1). All these findings supported a diagnosis of deep neck infection with abscess formation. The patient underwent surgical incision and drainage of the deep neck abscess. Systemic antibiotics were administered, and the wound was irrigated every 6 h. However, at 5 days after treatment, the festering wound had not improved, and a second operation was immediately performed. During surgery, a residual abscess was discovered in a deep neck space. Therefore, we employed an innovative continuous-irrigation approach for wound care after surgery using a double-lumen tube consisting of an inlet tube and an outlet tube (Fig. 2). Saline water was continuously injected through the inlet (irrigation) tube and suctioned from the outlet (draining) tube. After 5 days of intensive irrigation, wound swelling and discharge was considerably reduced and the wound had been closed. The patient was subsequently discharged from the hospital, and no recurrence was noted at the 6-month postoperative follow-up.

## 3 Discussion

This is a case of deep neck infection that was successfully treated using an innovative continuous-irrigation approach for wound care after surgery. On the basis of our experience, we connected a 500-cc intravenous bag of 0.9 % normal saline to the extension tube. We set the flow rate of the input infusion tube to 40–50 gtt/min (equal to 120–150 mL/h), and one 500-cc intravenous bag was administered in 3–4 h. The output cannula was connected to a suction bottle through an extension tube; thus, negative pressure was maintained to evacuate dirty fluid and debris from the wound. We recommend the use of at least three bags per day depending on wound assessment. In the interval between irrigations, patients should attempt to leave their beds and move around; they should also obtain a full night’s rest without disruption.

Despite the convenience of our novel irrigation system, it is not suitable in the following two clinical situations: (1) patient agitation—patient does not cooperate by lying still; (2) neck fistula connecting to the oral cavity, pharynx, larynx, or trachea—here, the risk of choking is increased due to flushing into the respiratory tract.

This approach exhibited several advantages. First, compared with intermittently manual irrigation, a continuous-irrigation device can more effectively keep a wound clean. Second, the automated design of this device can also considerably reduce the workload for clinical staff and is especially beneficial for understaffed institutions. Third, our device does not require expensive materials or complex technology.

## 4 Conclusion

In conclusion, we consider this innovative continuous-irrigation approach to be an alternative approach for wound care in patients with deep neck infection.

## Declaration of Competing Interest

The authors report no declarations of interest.

## Funding

The authors received no financial support for the research, authorship, or publication of this article.

## Ethical approval

The Institutional Review Board of Chang Gung Memorial Hospital approved this study (CGMH-IRB No. 202002100B0).

## Consent

There are no identifying details in this study. Patient has provided informed consent for publication of the case.

## Author contribution

Ming-Shao Tsai performed the operation. Meng-Chang Ding and Chih-Yuan Lee prepared the manuscript. All authors performed perioperative and postoperative therapy and approved the final version of the manuscript.

## Registration of research studies

Not applicable.

## Guarantor

Dr. Ming-Shao Tsai.

## Provenance and peer review

Not commissioned, externally peer-reviewed.
